# Perioperative Sleep Disorder: A Review

**DOI:** 10.3389/fmed.2021.640416

**Published:** 2021-06-07

**Authors:** Dandan Lin, Xiao Huang, Yi Sun, Changwei Wei, Anshi Wu

**Affiliations:** Department of Anesthesiology, Beijing Chao-Yang Hospital, Capital Medical University, Beijing, China

**Keywords:** perioperative sleep disorder, mental diseases, perioperative neurocognitive disorder, post-operative pain, dexmedetomidine

## Abstract

Patients in the perioperative period usually present with different types and degrees of sleep disorders, which can severely affect their post-operative outcomes. Multiple risk factors may lead to the occurrence of perioperative sleep disorders, including personal factors, psychological factors, surgery factors, and environmental factors. In this review, we summarize the potential risk factors for perioperative sleep disorders during hospitalization. And it also provides an overview of perioperative outcomes and potential therapeutic prevention of perioperative sleep disorders. However, the further search is necessary to investigate the effectiveness and safety of preventions in the clinical practice and push forward the therapies.

## Introduction

Sleep is one of the human basic physiological needs. Sleep disorders, which can occur short term or long term in the perioperative period, affect a large number of patients undergoing surgery. Sleep disorders can adversely affect patient recovery, increase the incidence of post-operative neurological outcomes and pain, and decrease hospitalization satisfaction. Despite the considerable threat pose to public health, sleep disorders are poorly understood, underdiagnosed, and poorly managed, especially in perioperative patients. There is unavoidable heterogeneity of methodological and characteristics of the population among studies. The potential negative consequences of sleep disorders indicate a need to pay more attention to their prevalence in a surgical population.

**Figure 1:**
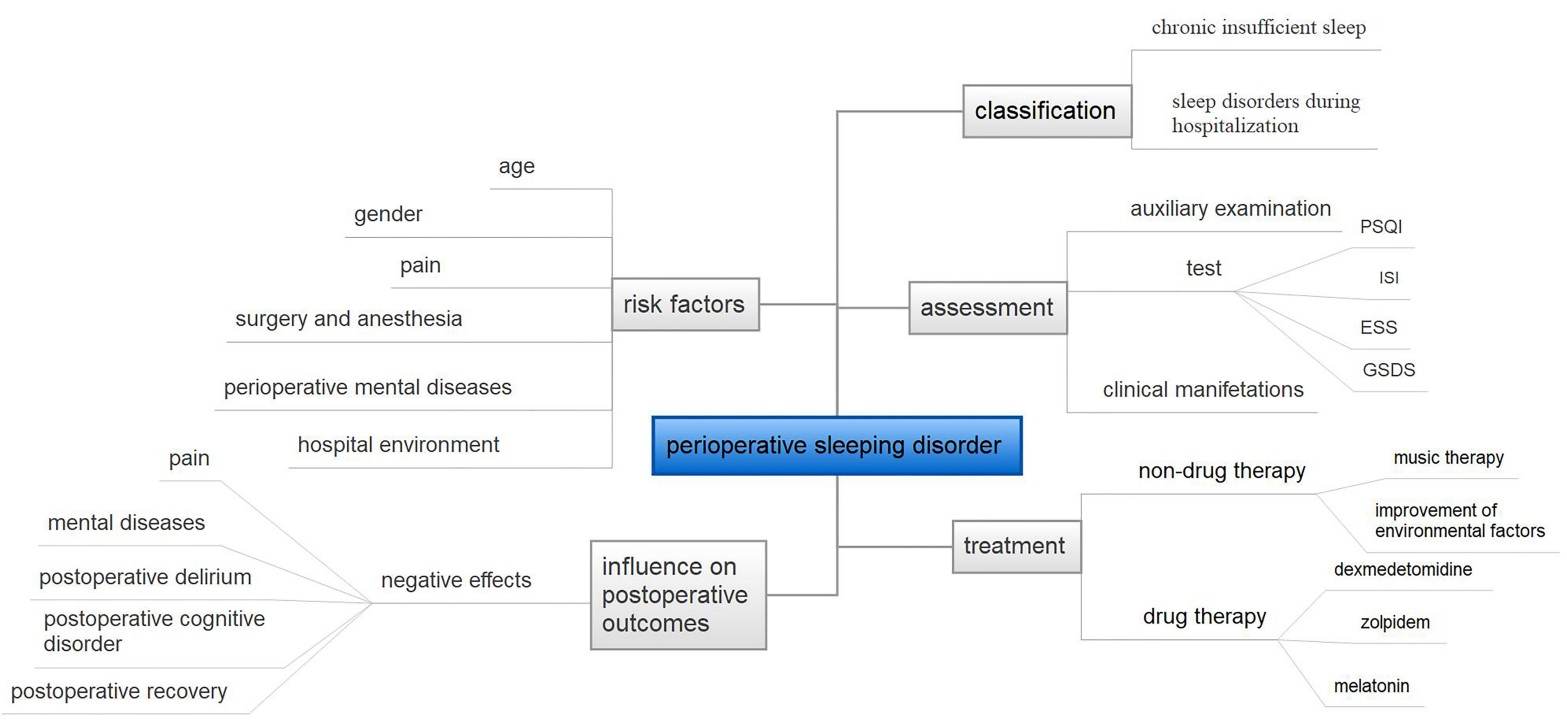
The overview of this paper. PSQI, Pittsburgh Sleep Quality Index questionnaire; ISI, Insomnia Severity Index; ESS, Epworth Sleepiness Scale; GSDS, General Sleep Disturbance Scale.

However, the high incidence of perioperative sleep disorders and the detrimental effect on post-operative recovery make a more in-depth study of perioperative sleep of great value. We summarize the literatures in recent years. This review describes the diagnosis, prevalence, classification of sleep disorders, and their effects on patients' post-operative outcomes (in Figure 1). We discuss the potential risk factors of perioperative sleep disorders throughout patients' hospitalization. Furthermore, the discussion concludes potential therapeutic intervention which can be applied to clinical practice in the future.

## Perioperative Sleep Disorder

### Incidence

Sleep disorders are prevalent in perioperative patients. About 8.8–79.1% of patients suffered from sleep disorder before the surgery (1–3). The sleep disorder may last for a long time after surgery. Halle reported that 49.7% of patients undergoing lung cancer surgery had sleep disturbance at 1 year post-operatively (3). And patients in the intensive care unit after thoracic surgery reported worse sleep quality, with 62% of the patients at 6 months and by 12% of the patients at every follow-up visits time points suffering from poor sleep (4). Moreover, the incidence of sleep disorder is different among different types of surgeries and diseases. Preoperative sleep disturbance is common in patients with rotator cuff tears, but sleep quality improves significantly after surgery (5). However, in arthroscopic hip surgery, 8.8% had a diagnosis of insomnia before surgery and 17.8% after surgery (6). The higher incidence of insomnia after surgery may be due to pain, opioid use, etc. Besides, the occurrence of sleep disorders was always neglected by medical staff. For example, nearly a quarter of cardiac surgery patients were hospitalized with obstructive sleep apnea, where 80% of these cases were undiagnosed before surgery. The deep hypoxic injury caused by apnea after surgery is often misdiagnosed as cardiac arrest due to other causes (7).

Patients have a particularly high incidence of post-operative sleep disturbance, while preoperative sleep disturbance also predicts opioid use (6). Therefore, it is of great significance to evaluate the sleep status of patients, screen out the patients with sleep disorders, and give appropriate treatment in clinical work.

### Assessment

The assessment of sleep disorders is mainly based on clinical manifestations (difficulty falling asleep, early awakening, night terrors, nightmares, or abnormal behaviors during the sleep period) and auxiliary objective indicators (in Table 1). Subjective sleep quality assessment is mainly measured by the scales, the Pittsburgh Sleep Quality Index questionnaire (PSQI), Insomnia Severity Index (ISI), Athens Insomnia Scale, Epworth Sleepiness Scale (ESS), General Sleep Disturbance Scale (GSDS). In clinical studies, the most commonly used assessment is PSQI. PSQI has high reliability and validity. PSQI could evaluate seven areas of sleep, which include subjective sleep quality, sleep latency, sleep duration, habitual sleep efficiency, sleep disturbances, use of sleeping medication, and daytime dysfunction over the last month. The severity of sleep quality is based on the score of 19 items in the above seven areas. The higher scores indicate worse sleep. However, the PSQI is used to evaluate sleep quality in 1 month. As another assessment of sleep quality, ISI is a self-rating scale measuring insomnia symptoms and consequences. The items were designed to assess the severity of sleep-onset, sleep maintenance difficulties, satisfaction with current sleep pattern, interference with daily functioning, noticeability impairment, and degree of distress or concern caused by sleep disorder (8). The severity of insomnia is also based on the total score of these items. In addition, ESS is used to assess the tendency to doze during the daytime (9).

**Table 1:** The common assessments of PSD.

**Assessment**	**Content**	
Subjective assessment	PSQI	Subjective sleep quality, sleep latency, sleep duration, habitual sleep efficiency, sleep disturbances, use of sleeping medication, and daytime dysfunction over the last month.
	ISI	The severity of sleep-onset, sleep maintenance difficulties, satisfaction with current sleep pattern, interference with daily functioning, noticeability impairment, and degree of distress or concern caused by sleep disorder.
	AIS	Sleep induction, awakenings during the night, final awakening, total sleep duration, sleep quality, well-being, functioning capacity, and sleepiness during the day.
	ESS	Daytime sleepiness.
	GSDS	Sleep onset latency, mid sleep wakes, early awakenings, quality of sleep, quantity of sleep, excessive daytime sleepiness, and medications for sleep
Objective assessment	PSG	Total sleep time, sleep onset latency, wake time after sleep onset, awakenings, amount and percentage of the sleep stages (non-rapid eye movement and rapid eye movement)
	Actigraphy	Set time in bed, sleep efficiency, total sleep time, sleep-onset latency, and wake after sleep onset

*PSQI, Pittsburgh Sleep Quality Index questionnaire; ISI, Insomnia Severity Index; AIS, Athens Insomnia Scale; ESS, Epworth Sleepiness Scale; GSDS, General Sleep Disturbance Scale; PSG, polysomnography*.

The scale measure of sleep was not related to objective sleep (9). Sleep is usually categorized into non-rapid eye movement sleep (NREM) and rapid eye movement sleep (REM). The NREM sleep is divided into stages N1, N2, and N3. Polysomnography (PSG), which can record electroencephalogram (EEG), electromyogram, electrocardiogram, and other vital signs, is used for assessment of sleep quality and sleep structure. The PSG is often used as an auxiliary diagnosis of sleep disorders and to detect the EEG changes in clinical studies. Actigraphy is also a reliable economical measurement of subjective sleep. It can evaluate set time in bed, sleep efficiency, total sleep time, sleep-onset latency, and wake after sleep onset (10). More validation tests are needed to evaluate the accuracy of actigraphy. Without a uniform standard, the perioperative sleep disorder diagnostic criteria vary from studies, where different classification systems, quantitative criteria to evaluate the sleep disorders independently. Sometimes other criteria such as face-to-face or telephone follow-up, survey and sleep diary, time of the assessment, and population characteristics, types of co-morbidities, and surgery are used as well.

### Classification of Sleep Disorder

In general, sleep disorders in the hospitalized patient can be divided into two main categories: chronic insufficient sleep and sleep disorders during hospitalization. According to the International Classification of Sleep Disorders, version 3 (ICSD-3), sleep disorders can be categorized based on clinical symptoms (11, 12). Sleep disturbances include insomnia, sleep-related breathing disorders, central disorders of hypersomnolence, sleep-related movement disorders, circadian rhythm sleep disorders, parasomnias, physiological (organic) sleep disorder, other sleep disorder not due to a known substance or physiological condition, environmental sleep disorder, etc. (13–16). As there are a variety of sleep categories, here we introduce two common clinical types.

#### Insomnia

Insomnia is the most common type of sleep disorder, in which people experience difficulty in sleep initiation, maintaining, consolidation, and poor sleep quality. The sleep disorder is chronic if it last for more than 3 months, while a short term sleep disorder lasts for <3 months. Insomnia is associated with multiple factors, such as genes, environment, psychology. It is the most common type of sleep disorder. Insomnia is a common sleep problem that is particularly common in perioperative patients (17, 18).

#### Sleep-Related Breathing Disorders

Sleep-related breathing disturbance includes obstructive sleep apnea disorders (OSAS), central sleep apnea disorder, sleep-related hypoventilation disorders, and sleep-related hypoxemia disorder. Of these, OSAS is one of the most common types in the perioperative period. OSAS is a clinical syndrome with a series of pathological changes caused by recurrent pauses in breathing during sleep, which results in hypoxia and carbon dioxide buildup in the body. It not only disrupts sleep but also leads to drowsiness, systemic hypertension, diabetes, cardiovascular events, etc., which could seriously affect patients' quality of life and cause social problems (19). A meta-analysis showed that the presence of OSA is associated with an increased risk of post-operative complications (20). These patients are more likely to have comorbid post-operative dyspnea and higher pain scores (21). Patients with combined obstructive sleep apnea have significantly increased post-operative risks such as pulmonary complications and co-morbidities (20).

## Risk Factors Of Perioperative Sleep Disorder

### Age

Sleep patterns and quality are known to change throughout a person's life. A study showed older subjective age was related to worse sleep (22). Changes of sleep-wake physiology in aging adults have been well-characterized by polysomnography (23, 24). Sleep disturbance occurs commonly in aging adults, including a decline of total sleep time, sleep efficiency, an increase in time awake after sleep onset, and sleep latency (25). And the percentage of time in REM sleep and N3 sleep are also lower in aging adults (26). The aging process may result in multiple organ senescence and pathological changes, including joint degeneration, hypertension, diabetes, cancer, etc. The pain and mental diseases caused by these pathological changes can also affect the sleep (27, 28). In addition, the older patients experience more difficulties adapting to new environment in pre-operative days in the hospital, which would aggravate the sleep disturbance.

### Sex

Perioperative sleep disturbance has a gender difference. Women were reported to experience more sleep problems than men, and the incidence of insomnia in women was 1.5 times higher than that in men (29). This difference could be associated with socioeconomic factors, physiological factors, and psychological factors (30). Several studies have indicated that female reproductive hormones may change sleep pattern, where an increase in estrogen could increase REM sleep time and decrease REM latency (31, 32). Females are also more prone to be exposed in a more stressful situation and has more difficulties problems in the preoperative period (33). In a power spectral analysis, electroencephalographic differences of insomniac men and women were observed. The female with insomnia had increased beta 2 power and men with insomnia had reduced alpha power throughout night (34).

### Pain

Pain is the major risk factor for sleep deprivation both in the perioperative period. Perioperative pain is always often by physical disease, especially in patients with arthrosis diseases. About 50% of patients with insomnia experienced chronic pain (35). Moreover, sleep deprivation and pain feed each other in a vicious. Sleep deprivation may lead to hyperalgesia and increased the onset of chronic pain, pain severity, and durations (36). And the pain may aggravate sleep deprivation. Medication for severe pain, like opioids, has a cofounding negative effect on sleep (37).

### Surgery and Anesthesia

Severe sleep deprivation was found in patients undergoing surgery. Prevalence of sleep disorders varied across studies due to different diagnostic criteria, the surgery type, and follow-up time. In total knee arthroplasty, sleep disturbances persisted for 2 months after surgery, with primary insomnia in 75.9% of patients and secondary insomnia in 24.1% of patients (38). Patients are reported to experience shorter total sleep time, fragmented sleep, decrease in REM and N3 after surgery (39). Different types of surgery have varied effects on patients' sleep quality. The patients reported more waking up times after orthopedic surgery because of the possible increased level of pain. Moreover, the major surgery has greater suppression of REM sleep (40). The patients might have worse quality of sleep, which was related to the bigger operative trauma (41).

General anesthesia can disturb post-operative sleep patterns by affecting the sleep-wake cycle. This may be because EEG features of the anesthetics were partially similar to that of sleep, like slow-delta oscillations and gamma oscillations (42). Different anesthetic medications and managements result in different changes in sleep patterns. Opioids are wildly used in analgesia, including remifentanil, sufentanil, and fentanyl. For example, some of the effects of the opioid which could last after surgery and post-operative analgesia may influence the first-day sleep after surgery. During the anesthesia period, ketamine, or propofol infusion had the opposite effect ketamine enhanced wakefulness and inhibited NREM sleep, while propofol inhibited wakefulness and enhanced NREM sleep (43).

### Perioperative Mental Diseases

The perioperative mental diseases include general anxiety disorder, major depression, dysthymia, and schizophrenia, which commonly occur in the perioperative period (44). In our previous study, the incidence of preoperative anxiety and depression were 23.4 and 21.5% in patients undergoing none-cardiac surgery respectively. In patients undergoing abdominal aortic aneurysm repair surgery, the prevalence was up to 21.2–29.3% and 28% (45). During the preoperative period, the risk factors for anxiety and depression include waiting for hospitalization, fear for death, preoperative pain, cancer, potential risk for surgery, and post-operative recovery (46, 47). Sleep disturbance is a major symptom and complication of most mental diseases (27). Patients with depression showed a decreased slow-wave sleep and disinhibition of REM sleep both in REM density and total REM sleep time (48). And the preoperative mental diseases also correlate with post-operative pain. The patients with preoperative anxiety and depression had higher VAS scores after surgery and long-term pain, which might lead to sleep deprivation (49). Perioperative mental diseases, sleep disturbance, and post-operative pain can create a vicious circle (27).

### Hospital Environment

The hospital environmental factors also influence the quality of sleep. A study showed that 36% of hospitalized patients reported new-onset of insomnia, and 10% of which were clinical insomnia and severe insomnia. The symptoms could persist a short time after being discharged from the hospital (50). Environmental factors including noise, lights in the ward led to sleep deprivation in orthopedic patients after surgery (51). The hospital noise was caused by the medical staff's conversation, alarm of medical monitor, and interactions from other patients. And the circadian rhythms are regulated by daylight and has influences on the molecular biology (52). For example, the morning light was too strong to maintain the secretion of melatonin which correlated with high-quality sleep in ICU. With the need for medical care, impropriate light may also disturb the patient's sleep at night.

## The Effects Of Perioperative Sleep Disorder On Patients' Post-Operative Outcomes

Sleep deprivation can negatively impact patient prognosis, physiological function, and hospitalization satisfaction. Clinicians need to identify sleep disorders as early as possible and provide appropriate assistance to patients (53). Sleep disorders in patients undergoing surgery have a sophisticated, multi-factorial effect on pain, fatigue, and depression.

### Pain

There is an interaction between pain and sleep. Pain may exacerbate sleep disturbances. At the same time, sleep disorders can in turn worsen the pain. For surgical patients, the interaction between sleep disturbance and pain in the perioperative period is more complex. Sleep disturbance has become a significant predictor of post-operative pain (54). Previous research indicated that breast cancer patients with bad pre-surgical sleep had worse post-operative pain (1). Pre-operative insomnia severity was significantly correlated with post-operative pain. Post-operative pain was the main reason for interrupted sleep in about 48 patients (51). Besides, pre-operative sleep efficiency was positively associated with patient satisfaction (55). This may attribute to short-term sleep disturbances that can aggravate post-operative pain hypersensitivity (56). In a pilot study of patients undergoing joint replacement, an extension of preoperative sleep reduced both pain and opioid consumption during the first 3–4 post-operative days (57). Current literature is largely supportive of perioperative sleep disturbance as a risk factor for post-operative pain, but the specific mechanisms are not fully elucidated. A conjecture is that perioperative short-term sleep deprivation may increase the expression and activity of L-type calcium channels in the lumbar dorsal root ganglion, delaying recovery from post-operative pain, and blocking these channels decreases the effects of sleep deprivation (58).

### Mental Diseases

The mechanisms of perioperative sleep disorders are not yet to be fully discovered because sleep behavior vary from country to country. Ongoing sleep disruptions and sleep disturbances may evolve into depression. There is also an interrelationship between insomnia and depression, with high rates of insomnia and sleep disorders in depressed populations, and insomnia is a strong predictor of the development of depression. According to research, most psychiatric patients have some kinds of sleep disorders (59). An increased proportion of post-operative mental health disorders usually accompanies a higher rate of sleep disturbances (60). Sleep difficulties can lead to non-fatal self-harm and suicide in patients after bariatric surgery (61). Sleep disturbances both a risk factor and a clinical manifestation of mental illness. However, their interactions are complex and there is little literature has been done to illustrate the specific mechanisms of action (62).

### Post-operative Delirium

According to the results of a meta-analysis, pre-existing sleep disturbances may be associated with post-operative delirium (POD) (63). A study about cardiac surgery found that pre-operative sleep disorder was significantly associated with POD and was the main predictor of POD (64), especially in older individuals (65). The risk of post-operative delirium is significantly higher in older adults with chronic sleep disturbance before hospitalization than in those without sleep disorders. Sleep interruptions in the hospital may further increase this risk (66). A possible explanation is as follows: OSA may cause central neuronal apoptosis, and decrease activity in multiple brain regions (67). And as a result, dysfunctional connectivity of different brain areas by the circadian clock can cause symptom fluctuations and sleep-wake cycle disorders in delirium (68). While most studies have suggested a strong correlation between delirium and sleep disorders, one study proposed the opposite opinion. It concluded that there was no significant correlation between obstructive sleep apnea and post-operative delirium in the routine care of the intensive care unit (69). Therefore, the relationship between sleep disorders and delirium remains to be unclear. More in-depth research in this area is needed in the future.

### Post-operative Cognitive Disorder

The mechanism of insomnia and cognitive impairment has been investigated recently, and the results showed that the link between insomnia and cognitive impairment was explained by brain. There was some proof for the mechanism of insomnia and cognitive impairment. In addition to its negative association with mental health, insomnia can lead to negative cognitive development. However, many studies have reported negative correlations between insomnia type traits and cognitive performance. It was shown in an experiment that pre-operative sleep disturbances may exacerbate post-operative cognitive impairment in elderly mice by exacerbating surgically induced neuroinflammation, nerve damage, and disruption of the blood-brain barrier (70). However, isoflurane-induced cognitive impairment in elderly mice can be prevented by preoperative pharmacological improvement (71). Resynchronization of circadian rhythms and improved sleep quality may be one of the crucial factors in the prevention or treatment of POCD (72). Patients who develop POCD after major abdominal surgery have poor sleep quality and significantly more frequent night wakings. It is likely that disturbed sleep and circadian rhythms may be the basis for cognitive dysfunction after major surgery (73). Pharmacological treatment of sleep disorders may be a potential future direction for the prevention and treatment of POCD (74).

### Post-operative Recovery

Sleep-disordered breathing is a possible independent predictor of emergence agitation in pediatric ambulatory surgery (75). It was reported that surgery in the morning or at night has different effects on post-operative sleep. The incidence of post-operative sleep disturbance was significantly increased in patients who had surgery at night, along with an increase in both post-operative adverse effects and pain (76). The reason may be that physiological changes in the body at different times of the day affect the anesthetic drug interaction and metabolic response.

We note that some surgeries can improve sleep disturbance in patients. The percentage of patients undergoing primary total joint replacement who need to seek sleeping help decreased from 70.2% at preoperative baseline to 44.7% late in the post-operative period (77). Female incontinence surgery which significantly improves quality of sleep. For instance, women with urinary incontinence showed effective sleep improvement after surgery (78). Another study had similar findings. Patients with femoroacetabular impingement syndrome had a high incidence of pre-operative sleep disturbance. These patients had an effective early improvement in sleep disturbance after arthroscopic hip surgery (79).

## Treatment Of The Perioperative Sleep Disorder

### Non-drug Therapy

Non-drug therapy refers to the treatment of the perioperative sleep disorder using environmental elements. For example, music therapy used prior to operation can relieve the anxiety and stress from patients (80). Music therapy was thought to be effective for primary insomnia. A study of patients undergoing cardiac surgery found a 30 min music therapy 1 night before the surgery might improve subjective sleep quality and prolong the sleep duration (81). For older patients in ICU, single music therapy improved subjectively sleep quality in post-operative day 2. An elevated saliva melatonin was observed in the music therapy group with a negative statistical result (82). This indicated that music therapy might influence the secretion of melatonin and cortisol. Furthermore, improvement of environmental factors, such as single bed ward was also showed improvement of perioperative pain and post-operative outcomes.

Preoperative anesthetic clinic has been shown to reduce the stress and anxiety of waitlisted patients with severe complications of diseases. The talks with patients make them clear about the potential risk for the surgery and anesthesia. The anesthetic clinic decreased the incidence of preoperative anxiety (46).

### Drug Therapy

#### Dexmedetomidine

Dexmedetomidine, a highly selected a-2 adrenoceptor agonist, is commonly used in perioperative sedation and analgesia. Continuous infusion of dexmedetomidine nocturnal in ICU patients prolonged the total sleep time, increased sleep efficiency, and quality (39). An infusion of dexmedetomidine during the operation could also improve post-operative sleep quality and decrease severe sleep disturbance (83). A real-world cohort study showed that patients undergoing gynecological, urological, and orthopedic spine surgery experienced more improvement on the quality of sleep (84). This is particularly true for patients with higher risk of sleep disturbance in such surgery and female patients. The mechanisms of dexmedetomidine is yet to be completely understood. The intraoperative use of dexmedetomidine may not improve post-operative pain or post-operative analgesia, which was one of the most important risk factors of post-operative sleep quality. Thus, the improvement of subsequent sleep quality might be correlated with other factors. In a study that reported the complete polysomnogram recordings of patients undergoing non-cardiac surgery, the percentage of N2 sleep was 15.8% in the placebo group and 43.5% in the dexmedetomidine (39). Sedation with dexmedetomidine was closely resembling physiological N2 sleep. Compared with the control group, dexmedetomidine infusion may increase non-REM 2 sleep and decrease REM sleep (85). The Electroencephalogram (EEG) was used to test sleep spindles, which are considered sleep maintaining events, in dexmedetomidine sedation period and normal physiological sleep. The spindle density, amplitude, and frequency content in dexmedetomidine EEG recording were not different from that in physiological sleep (86). Cai reported that a low dose of dexmedetomidine (0.1–0.2 ug/kg/h) had a better improvement of post-operative sleep quality than a high dose of dexmedetomidine (>0.2 ug/kg/h) (87). The optimal concentration during the short term and long term after surgery deserves further studies.

#### Zolpidem

Zolpidem is a non-benzodiazepine drug, which can improve post-operative sleep without serious aberrant side effect. It is used for the treatment of insomnia and repairing disrupted sleep by improving the proportion of REM and SWS sleep. Patients receiving zolpidem 2 days pre-operative to 5 days post-operatively improved perioperative sleep experience and satisfaction, reduced the level of perioperative anxiety, and depression (88). However, there is an evidence that zolpidem prescription was associated with an increased risk of suicide attempts. In a nationwide population study in South Korea, the incidence rate ratio of suicide was 70.6% at 2 days before zolpidem prescription and decreased to 63.35% at 1 day after zolpidem prescription in the suicide attempt group. This indicated that zolpidem didn't contribute to an additional increase in suicide attempts (89).

#### Melatonin

Melatonin, synthesized, and secreted by the pineal gland, is an endogenous hormone that can regulate the circadian rhythm. The melatonin application in the perioperative period can improve sleep quality without apparent side effects. Madsen reported oral melatonin (6 mg) 1 h before bedtime could improve sleep efficiency and reduce wake duration after sleep onset after surgery in breast cancer surgery (90). In a small sample size randomized clinical study, the melatonin could decrease the duration of sleep latency, daytime naps, and night awakening after surgery (91). The total sleep duration was increased in post-operative days 1 and 2. The VAS score decreased in the melatonin group. The mechanism of melatonin application is yet to be discovered. Jia found that melatonin treatment was only reflected in increase of the quantitative delta power in the dark phase. However, Jia argued that it had no improvement in sleep quality (92). Melatonin microinjected to the perifornical lateral hypothalamus at dark onset could significantly increase NREM sleep and reduce wakefulness (93). In addition, melatonin is considered a therapeutic substance for post-operative delirium. In a meta-analysis that included 6 studies, the perioperative melatonin could reduce the incidence of delirium in older patients (94). One study found that for patients undergoing bariatric surgery, melatonin could improve post-operative recovery (95). Besides, some small sample size research found the improvement of anxiety and depression symptoms after using melatonin. Hanse reported that the incidence of depression symptoms were lower after administration of melatonin 12 weeks post-operatively (96). Melatonin may improve the recovery quality of patients through improving sleep and regulating circadian rhythms.

## Conclusion

With the development of perioperative medical care, sleep quality, and sleep patterns caused increasing attention. Multiple factors might contribute to perioperative sleep disorder, including age, perioperative mental diseases, surgery, anesthesia, and environmental factors. Given the high prevalence of sleep disturbances in patients in the perioperative period and the significant impact on prognosis, it is crucial to optimize patients' perioperative sleep. A multimodal approach of pre-operative counseling, early post-operative sleep modification, and some medication use may improve transient sleep disturbances in surgical patients. Patients' perioperative sleep disturbances are multifactorial and interdependent on pain, fatigue, and depression. Future studies need to use larger, more homogeneous patient populations which will be essential to fully understand the mechanism of sleep disorders and disease and surgery and to develop accurate and effective protocols for the treatment of sleep and disease.

## Author Contributions

DL and XH contributed to conception and design of the review. YS, DL, and XH searched the database and wrote the first draft of the manuscript. CW and AW wrote sections of the manuscript. All authors contributed to manuscript revision, read, and approved the submitted version.

## Conflict of Interest

The authors declare that the research was conducted in the absence of any commercial or financial relationships that could be construed as a potential conflict of interest.
